# Angiotensin II related glial cell activation and necroptosis of retinal ganglion cells after systemic hypotension in glaucoma

**DOI:** 10.1038/s41419-022-04762-4

**Published:** 2022-04-09

**Authors:** Soo Ji Jeon, Jungbin Huh, Eojin Jeong, Chan Kee Park, Hae Young Lopilly Park

**Affiliations:** 1 Department of Ophthalmology, Bucheon St. Mary’s Hospital, College of Medicine, The Catholic University of Korea, Seoul, Republic of Korea; 2 Department of Medical Life Sciences and Department of Biomedicine & Health Sciences, College of Medicine, The Catholic University of Korea, Seoul, Republic of Korea; 3 Department of Ophthalmology, Seoul St. Mary’s Hospital, College of Medicine, The Catholic University of Korea, Seoul, Republic of Korea

**Keywords:** Neuro-vascular interactions, Experimental models of disease

## Abstract

The purpose of this study was to design an animal model mimicking glaucoma with hemodynamic instability and to identify involvement of angiotensin II (AngII) and associated changes of the retina. Systemic hypotension was induced in Sprague–Dawley rats by oral hydrochlorothiazide administration. Rats were sacrificed at 4, 8, and 12-week time points. AngII and receptor levels were examined in the serum and retina. To examine the relationship between glia activation and associated RGC death, biochemical analysis of GFAP, Iba-1, and necroptosis associated factors such as TNFα, receptor-interacting protein (RIP) 1, 3, and inactive caspase 8 were explored. To investigate the difference in RGC death mechanism, JNK inhibitor or RIP3 inhibitor were given intraperitoneally to rats with ocular hypertension and systemic hypotension both to identify the pathway mainly involved. AngII and receptors were increased in the serum and retina of systemic hypotensive rat. At 4, 8, and 12 weeks after hypotension induction, glial activation was increased as indicated by GFAP and Iba-1 staining. TNFα, RIP3 were elevated. and downregulation of inactive caspase 8 was apparent in the retina of hypotensive rat. Electron microscopy revealed that necroptosis of RGC was gradually increased after systemic hypotension. Following intraperitoneal JNK inhibitor or RIP3 inhibitor administration, RGC loss was attenuated in systemic hypotensive rats but not in ocular hypertensive rats. In conclusion, AngII is involved in glial activation and associated RGC necroptosis following systemic hypotension. This pathway represents a novel and distinct cell death mechanism when compared to that involved in elevated intraocular pressure.

## Introduction

Glaucoma is characterized by progressive loss of the retinal ganglion cells (RGCs). Impaired aqueous humor outflow and subsequent elevation of intraocular pressure (IOP) are the main cause of RGC loss in glaucoma [1]. However, we clinically observe glaucoma patients with normal IOP and glaucoma patients that show progression even after sufficient IOP reduction with treatment [2, 3]. Therefore, investigating the cause and progression factor contributing to glaucoma other than IOP is important in preventing blindness from glaucoma. According to several studies, systemic vascular dysregulation is one important feature in glaucoma other than elevated IOP [4–6]. Systemic arterial hypotension, orthostatic hypotension, unstable or fluctuating systemic blood pressure (BP), Raynaud’s phenomenon, and migraine are common findings in glaucoma patients who were suspected to have systemic vascular dysregulation as a contributing factor in glaucoma [7–9].

The renin-angiotensin-aldosterone system (RAAS) is vital for the maintenance of arterial BP by extracellular fluid volume retention and systemic vasoconstriction [10]. Low arterial BP leads to an increased production of renin, which hydrolyzes the liver protein angiotensinogen to angiotensin I and II [11]. Recent reports have demonstrated that increased angiotensin II (AngII) levels induced glial inflammation in neural tissue such as the brain [12–14]. AngII receptors were also expressed in the retina, and increased AngII could activate retinal glial cells [15]. Activated glial cells could produce noxious materials including neuroinflammatory factors that could affect other neurons and result in neuronal cell death [16–18].

We hypothesized that RAAS could play a role in glaucoma patients without elevated IOP or contribute to glaucoma progression in glaucoma patients with controlled IOP. Especially, these glaucoma patients may have systemic vascular dysregulation and present low or unstable BP that may activate RAAS [19]. Currently, most laboratory studies related with glaucoma employ chronic ocular hypertension animal models with elevated IOP or injury models such as optic nerve crush [20], but these models have limitations in applying to glaucoma with normal range IOP. Therefore, we came up with a systemic hypotensive rat model that are characterized to have low and unstable BP using systemic hypotensive medication. Using this systemic hypotensive glaucoma model (termed the BP glaucoma model), we aimed to find out the distinct changes in the retina and RGCs with low or unstable systemic BP. At first, the changes in systemic level of AngII and angiotensin receptors in the retina were identified in the systemic hypotensive glaucoma model. Second, the glial cell activation and the type of RGC death were investigated in the retina. Third, its impact on cell death and related molecular pathway were investigated. Finally, a comparison between elevated IOP and systemic hypotension was conducted to investigate glial cell activation and related mechanisms.

## Materials and methods

### Animals

All experiments were performed in accordance with the Association for Research in Vision and Ophthalmology (ARVO) statement for the Use of Animals in Ophthalmic and Vision Research. We considered the National Institutes of Health Guide for the Care and Use of Laboratory Animals (NIH Publications, no. 80–23, revised 1996). All animals were cared for according to the regulations of the Catholic Ethics Committee of the Catholic University and the Institutional Animal Care and Use of Committee of the Catholic University of Korea. Total of 134 animals were used. Careful management of animals and procedures was ensured in order to minimize the number of animals used.

### Development of the systemic hypotensive rat

Adult male Sprague–Dawley rats (250–300 g) aged 7–8 weeks old were used in this study. Systemic hypotensive rat model was designed using hydrochlorothiazide (HCTZ) (Dichlozid; Yuhan, Seoul, Korea) to lower systemic BP without interfering with angiotensin pathways. Animals were randomly assigned to one of experimental or control group. We assessed dose-dependent responses with HCTZ and found an optimal dose that showed significant systemic hypotension when compared to controls. Systemic hypotension was induced by taking HCTZ 100 mg/kg dissolved in drinking water [21]. Systolic and diastolic BP were measured using a tail-cuff sphygmomanometer (Visitech BP2000, Visitech Systems, Apex, NC, USA) twice a day at the same time [22, 23]. Measurements were obtained every week throughout a 12-week experimental period. The mean value of the two measurements was calculated. Since the time and frequency of water drinking of individual rats were irregular, this may also induced BP fluctuation. Rats without a significant reduction in systolic BP < 120 mmHg or diastolic BP < 80 mmHg were excluded. In five rats in each group, BP measurements were performed throughout a day to obtain 24-h BP data. Measurements were performed every 3 h and twice at each time point (3, 6, and 9 AM and 12, 15, 18, 21, and 24 PM). The standard deviation of all measurement were calculated and the mean standard deviation of each group was regarded as BP fluctuation.

Blood samples from systemic hypotensive rats were centrifuged at 2000 xg for 10 min and stored at −20 °C. Serum concentrations of AngII were examined using ELISA kits (Ray Biotech, Inc., Norcross, GA, USA) according to the manufacturer’s instructions. Each well was prepared with 100 μl of anti-AngII antibody and incubated for 1.5 h at room temperature. After washing, 100 μl of samples was added to each well and incubated for 2.5 h at room temperature. Prepared streptavidin solution and 3,30,5,50-tetramethylbenzidine reagent were added continuously and the reaction was terminated by the addition of 50 μl of stop solution. The absorbance was measured at a wavelength of 450 nm.

### Fluorescein dextran angiography

A solution of fluorescein isothiocyanate-dextran (2 × 10^6^ molecular weight; Sigma-Aldrich) was prepared at a concentration of 10 mg/mL in PBS. After 4 and 8 weeks of systemic hypotension, three rats at each time points and control rats were anesthetized with 50 mg/kg of ketamine and 5 mg/kg of xylazine. Fluorescein isothiocyanate-dextran solution (0.5 mL) was injected into the tail vein of each rat, and a cover glass was placed on the cornea as a contact lens. Angiograms were established with a scan angle of 30°. Digital images at a magnification of x40 were captured using a charge-coupled device camera (DC500; Leica Microsystems, Heerbrugg, Switzerland) attached to a fluorescence stereomicroscope (MZ-III; Leica Microsystems, Wetzlar, Germany).

### Immunohistochemistry

After the rat sacrification, both eyes were enucleated and fixed in 4% paraformaldehyde at 4 °C for 10 min. The anterior segment of the eye was removed, and then, the posterior segment was fixed in 4% paraformaldehyde for 60 min and pre-embedded in 3% agar for cutting in the shape of a cube. Retinas in agarose cube were sectioned to a thickness of 50 μm with a vibratome. After washing several times with phosphate-buffered saline (PBS), non-specific binding was blocked with 10% normal donkey serum (NDS) in PBS for 45 min at room temperature. The slides were incubated overnight at 4 °C with primary antibodies. Mouse anti-Brn3a (brain-specific homeobox/POU domain protein 3a; Millipore, Billerica, MA, USA, 1:200), NeuN (neuronal nuclei; Millipore, 1:200), GFAP (glial fibrillary acidic protein; Millipore, 1:200) and Iba-1 (ionized calcium-binding adapter; 1:200) were used to observe RGC death and glial cell activation. To investigate angiotensin II receptor expressions, antibodies against angiotensin II receptor type 1 (AT-1) (1:100, Abcam) and type 2 (AT-2) (1:100, Abcam) were used. Primary antibody binding was detected by Alexa 488- and Alexa 546-conjugated secondary antibodies (Molecular Probes, Eugene, OR, USA). The slides were rinsed with 1× PBS and mounted with Fluoroshield mounting media including DAPI (Vector, Burlingame, CA, USA). Images of these stained tissues were acquired using confocal laser scanning microscopy (Carl Zeiss, Jena, Germany).

### Immunohistochemistry of flat-mounted retina

For the flat-mounted retina preparation, intact retinas were extracted and washed in PBS after fixation in 4% paraformaldehyde. Anti-Brn3a was used as the primary antibody for RGC labeling on retinal flat-mounts. Immunostained retinas were carefully flattened and mounted on microscopy slides with Fluoromount (Southern Biotech, Birmingham, AL, USA) and imaged using confocal laser scanning microscopy (Carl Zeiss, Jena, Germany).

Each flat-mounted retina was divided into four equal quadrants. For each retinal quadrant, three fields measuring 200 × 250 μm^2^ were randomly sampled from middle regions of the retina. The distance to the optic nerve from the corresponding field of each quadrant was equal. Labeled ganglion cells were counted at 200x magnification in twelve regions of each retina.

### Western blot analysis

Retinal sections were lysed in radioimmunoprecipitation assay (RIPA) buffer [50 mM Tris-HCl pH 7.5, 150 mM NaCl, 1 mM EDTA, 0.1% SDS, 1% IGEPAL and 0.5% sodium deoxicholate] containing protease and phosphatase inhibitor cocktails. Lysates were clarified by centrifugation at 10,000 xg for 25 min at 4 °C. Supernatants were assayed to measure protein content using a standard bicinchoninic acid (BCA) assay (Pierce, Rockford, IL, USA). Retinal extracts were separated by SDS-polyacrylamide gel electrophoresis and transferred onto a nitrocellulose membrane (Hybond-C, Amersham Pharmacia Biotech, Germany). Blots were stained with Ponceau S (Sigma) to visualize the protein bands and ensure equal protein loading and uniform transfer. The membranes were blocked for 45 min with 5% non-dried skim milk in Tris-buffered saline with Tween buffer (20 mM Tris-HCl pH 7.6, 137 mM NaCl, and 0.1% Tween 20). Then, the blots were probed for 24 h with antibodies against GFAP (Sigma), Iba-1 (Sigma), AT-1 (Abcam), AT-2 (Abcam), and GAPDH (Sigma). For necroptosis-related factors, antibodies against anti-goat TNFα (R&D systems), anti-rabbit RIP1 (Santa Cruz), RIP3 (Santa Cruz), and caspase 8 (Santa Cruz) were used. The blots were sequentially probed with horseradish-peroxidase (HRP)-conjugated goat anti-rabbit secondary antibody for 1 h at room temperature. Protein bands were detected using a chemiluminescence system (ECL, Amersham, MA, USA) and X-ray film. Relative intensity of the blots was measured using an ImageMaster VDS (Pharmacia Biotech, CA, USA), and the fold-changes in protein levels relative to GAPDH were calculated.

### Transmission electron microscopy (TEM)

Retinal tissues were fixed by immersion in Kamovsky’s solution for 24 h at 4 °C, and embedded in acrylic resin. Ultrathin sections (0.1 μm) were made, mounted on Formvar-coated slot grids, and stained with 3% lead citrate. The TEM images were acquired using Zeiss transmission electron microscope (Zeiss Inc., Thornwood, NY, USA). Approximately ten RGCs per sample were photographed and quantified for cell death pathways in a blinded manner. Cells with nuclear condensation and cellular shrinkage were defined as apoptotic RGCs, whereas cells associated with plasma and nuclear membrane discontinuities, dilation of perinuclear space, electron-lucent nucleus, and organelle swelling were defined as necrotic RGCs. Electrodense granular materials were defined as unclassified, because these materials could be subsequentness of both cell death pathways. The classification was performed by two blinded examiners (HYLP and SJJ), and disagreements were resolved by a third examiner (CKP).

### TUNEL staining

We detected apoptosis using In Situ Cell Death Detection Kit (Roche Applied Science) according to the manufacturer’s instructions. The retinas were dissected from the choroid, and the central portion of the superior nasal quadrant, 1.5 mm from the optic disc, was trimmed into small pieces. Cryosections of the retina (50 μm) were embedded in 4% paraformaldehyde and washed with PBS. The tissue was stained with the TUNEL method according to the manufacturer’s protocol (In Situ Cell Death Detection Kit; Roche Applied Science). The following day, after several washes with 0.1 M PBS, the sections were incubated with goat anti-rabbit Alexa 546 (Molecular Probes). After further washes in 0.1 M PBS for 30 min, the sections were mounted using VECTASHIELD Mounting Medium with DAPI (Vector Laboratories) and were examined by confocal laser scanning microscopy (Zeiss).

### Comparison with ocular hypertensive rat model

For comparison with ocular hypertensive rats, episcleral vein cauterization was performed. After intraperitoneal injection of 50 mg/kg ketamine with zolazepam (Zoletil; Virbac, Carros, France) and 15 mg/kg xylazine hydrochloride (Rompun, Bayer, Leverkusen, Germany), three episcleral veins were cauterized using a surgical microscope (Olympus, Tokyo, Japan). Retinal vascular perfusion was assessed using planar ophthalmoscopy after cauterization. IOP was measured using a Tono-pen (Solan, Florida, USA) after topical anesthetization with Alcaine (Alcon Laboratories, Fort Worth, Texas, USA).

### Statistical analysis

All data are presented as means ± standard deviation (SD). Two-sided Student’s *t*-tests were used to compare controls and experimental animals at each time point. *P* < 0.05 was considered statistically significant (*P* < 0.05 are indicated as ^*^, P < 0.01 as ^**^, and *P* < 0.001 as ^***^).

## Results

### Confirmation of the systemic hypotensive model and involvement of the RAAS

**Fig. 1:**
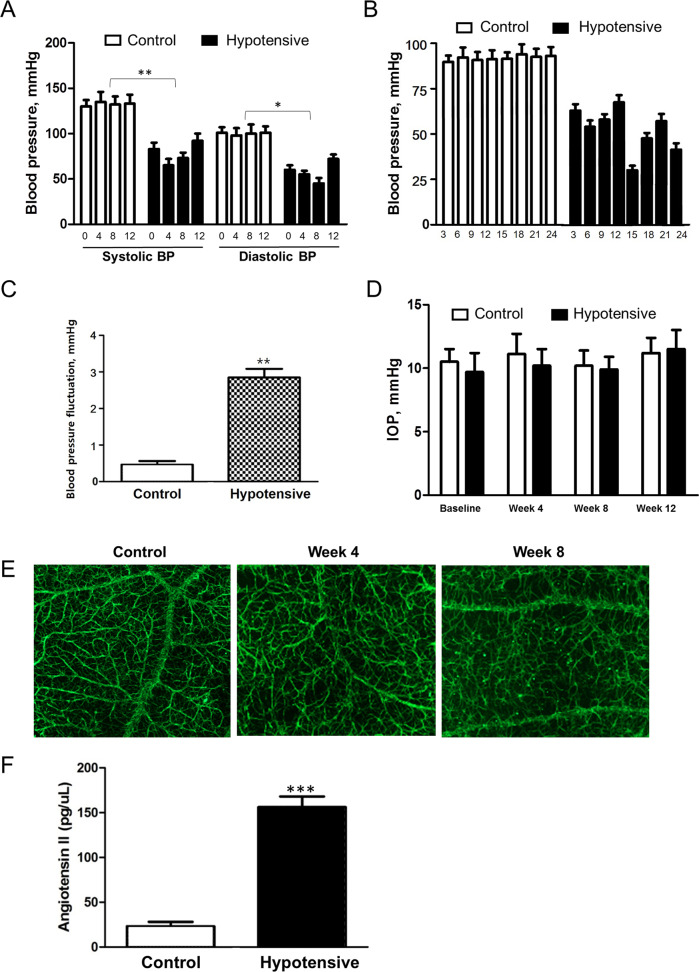
Profiles of systemic blood pressure and intraocular pressure and the level of serum AngII. **A** Systolic and diastolic BP of controls and systemic hypotensive rats. Rats administered with diuretics exhibited lower systemic BP than the controls. For BP measurements, *n* = 6 for control and *n* = 6 for systemic hypotension at each time period; total *n* = 12. Bar represents mean ± SD. Student’s *t*-test was used for statistical evaluation. *P < 0.05 compared to the control. **B** Twenty-four-hour BP measurements from five rats in each group. BP was lowered in hypotensive rats throughout the day. **C** The standard deviation of all 24-h BP measurements. The mean standard deviation was regarded as BP fluctuation, and the BP fluctuation from hypotensive rats was greater than controls. For 24-h BP measurements, *n* = 5 for control and *n* = 5 for systemic hypotension at each time period; total *n* = 10. Bar represents mean ± SD. Student’s *t*-test was used for statistical evaluation. *P < 0.05 compared to the control. **D** Intraocular pressure (IOP) of controls and systemic hypotensive rats. IOPs from both groups were not significantly different. For IOP measurements, *n* = 6 for control and *n* = 6 for systemic hypotension at each time period; total *n* = 12. Bar represents mean ± SD. Student’s *t*-test was used for statistical evaluation. *P < 0.05 compared to the control. **E** Fluorescein dextran angiography of rat retina. Gradual reduction in blood flow was observed in hypotensive rats. **F** Serum angiotensin II levels were elevated in systemic hypotensive rats. For the analysis of serum angiotensin II levels, *n* = 6 for control and *n* = 6 for systemic hypotension at each time period; total *n* = 12. Bar represents mean ± SD. Student’s *t*-test was used for statistical evaluation. *P < 0.05, **P < 0.01, and ***P < 0.001 compared to the control.

Animals administered with HCTZ exhibited lower and fluctuating systemic BP than the controls at the 4-, 8-, and 12-week time points. Tail-cuff-measured systolic BPs of the systemic hypotensive rats were 97.0 ± 13.5, 99.4 ± 12.6, and 100.1 ± 7.8 mmHg at 4, 8, and 12 weeks, respectively. The diastolic BPs of the systemic hypotensive rats were 58.0 ± 5.1, 52.4 ± 6.2, and 62.2 ± 8.5 mmHg at 4, 8, and 12 weeks, respectively. For the control group, systolic BPs at 4, 8, and 12 weeks were 137.2 ± 3.2, 136.8 ± 5.1, and 135.2 ± 6.1 mmHg, respectively. The diastolic BPs of the control group were 71.6 ± 8.1, 99.4 ± 12.6, and 100.1 ± 7.8 mmHg at 4, 8, and 12 weeks (Fig. 1A, all *P* < 0.001). Twenty-four-hour BP measurement were performed in each group, and we could observe that BP was lowered in the systemic hypotensive rats throughout the day (Fig. 1B). Additionally, the standard deviation of the 24-h BP measurements was greater in the systemic hypotensive rats indicating BP fluctuation in this group (Fig. 1C). IOP of the systemic hypotensive rats were within normal range and was not significantly different to that of control rats (10.4 ± 1.4 mmHg and 10.05 ± 1.6 mmHg in control and systemic hypotensive rats, respectively; *P* = 0.471) (Fig. 1D). To examine whether low or fluctuating BP is affecting the ocular circulation, we performed fluorescein dextran angiography. Although there was no vessel changes or vessel leak, gradual fluorescein filling defects indicating reduction in blood flow was observed in the retina of systemic hypotensive rats (Fig. 1E).

To examine the involvement of the RAAS after reduction and fluctuation in systemic BP, serum AngII levels were measured by ELISA. Serum AngII levels were elevated in the systemic hypotensive rat group (150 ± 14.5 pg/μL) when compared to the control group (31.8 ± 6.8 pg/μL; *P* < 0.001) (Fig. 1F). Therefore, we could confirm that reduced systemic BP induced systemic AngII level in this systemic hypotensive rat model.

### Changes in the retina of systemic hypotensive rat model

**Fig. 2:**
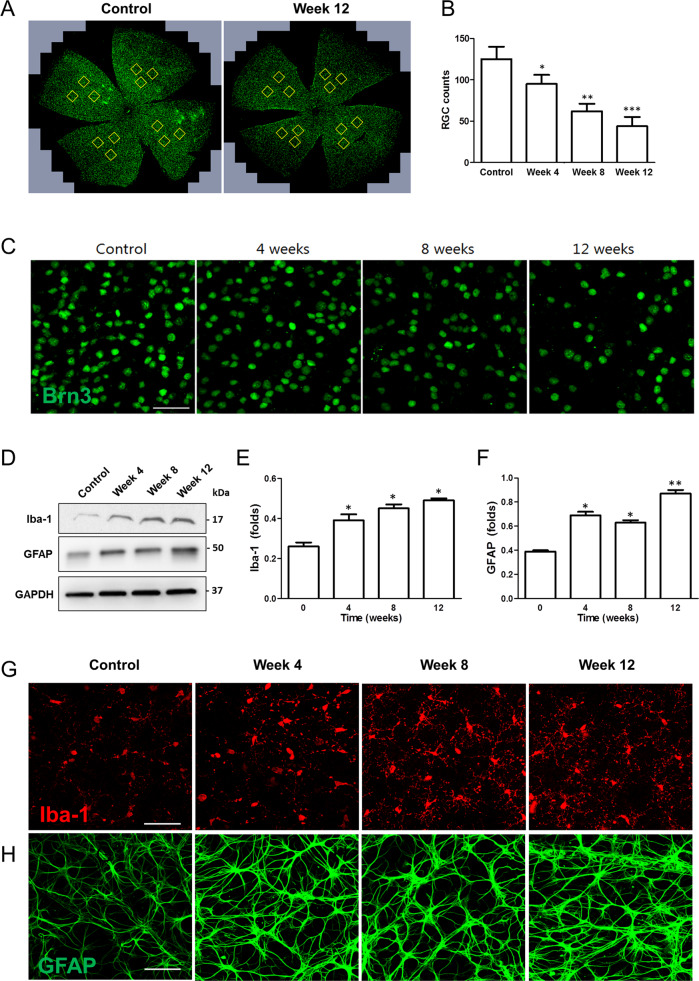
Changes of RGCs and glial cells in the retina after systemic hypotension. **A**–**C** Immunohistochemical staining of Brn3a from flat-mounted retina. Significant and gradual decrease of RGC number was revealed. For immunohistochemical staining, *n* = 6 for control and *n* = 6 for systemic hypotension at each time period; total *n* = 24. Bar represents mean ± SD. Student’s *t*-test was used for statistical evaluation. *P < 0.05 compared to the control. **D**–**F** Western blot and quantification of the blots. **G**, **H** immunohistochemical staining of Iba-1 and GFAP. Iba-1 and GFAP expression was increased in systemic hypotensive rats. GAPDH was used as an endogenous control in western blot analyses. Scale bars: 50 μm. For western blot analysis, *n* = 6 for control and *n* = 6 for systemic hypotension at each time period; total *n* = 48. Bar represents mean ± SD. Student’s *t*-test was used for statistical evaluation. *P < 0.05, **P < 0.01, and ***P < 0.001 compared to the control.

To examine changes of the RGCs in this systemic hypotensive rats, we performed immunohistochemical staining using flat-mounted retina (Fig. 2A). Analysis of the specific RGC marker, Brn3a, revealed a significant and gradual decrease in the number of RGCs throughout the 12-week experimental period following HCTZ intake when compared to the control (Fig. 2B, C).

We examined changes in Iba-1 and GFAP for detection of microglial and macroglial cell activation throughout the course of systemic hypotension. Western blot analysis of the retina of systemic hypotensive rat revealed a significant increase in Iba-1 and GFAP protein levels at weeks 4, 8, and 12 when compared to control (Fig. 2D–F). Therefore, RGC death and glial cell activation was observed in the retina of systemic hypotensive rat suggesting retinal changes related to systemic BP reduction and fluctuation. Immunohistochemical staining of the whole-mount retina of systemic hypotensive rat revealed increased Iba-1 and GFAP expression (Fig. 2G, H).

### Expression of AngII receptor type 1 (AT-1R) and type 2 (AT-2R) in the retina following systemic hypotension

**Fig. 3:**
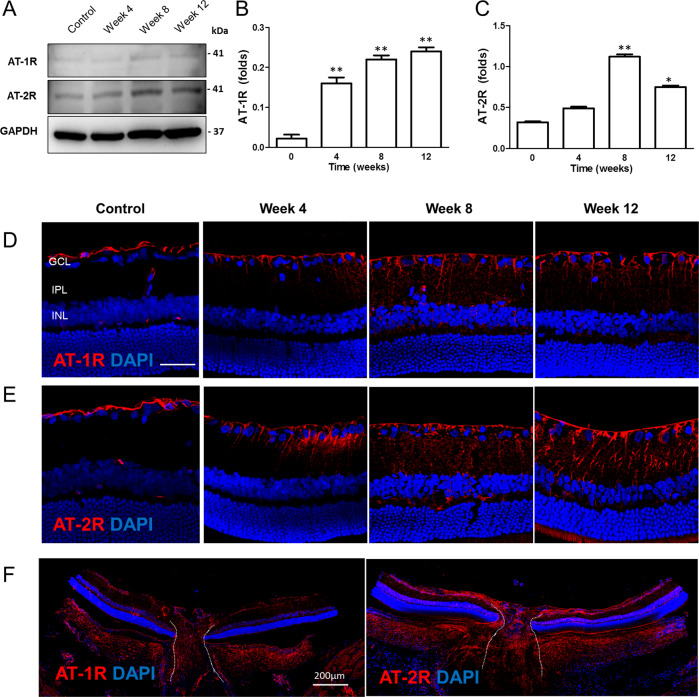
Changes of AngII receptors in the retina and optic nerve head after systemic hypotension. **A**–**C** Western blot of angiotensin II receptor type 1 (AT-1R) and type 2 (AT-2R) in the retina. GAPDH was used as an endogenous control. A significant increase of AT-1R and AT-2R was observed with the exception of AT-2R at week 4. For western blot analysis, *n* = 6 for control and *n* = 6 for systemic hypotension at each time period; total *n* = 48. Bar represents mean ± SD. Student’s *t*-test was used for statistical evaluation. *P < 0.05 and **P < 0.01 compared to the control. **D**, **E** Immunohistochemical staining of AT-1R and AT-2R in the retina and **F** optic nerve head. Increased expression of AT-1R and AT-2R was revealed throughout ganglion cell layer and inner plexiform layer of the retina, and the optic nerve head. Scale bars: 50 μm.

Alterations in AngII receptor (AT-1R and AT-2R) expression were identified in the retina after systemic hypotension. Western blot analysis revealed a significant increase in AT-1R and AT-2R protein levels at 4, 8, and 12 weeks following systemic hypotension, with the exception of AT-2R levels at week 4 (Fig. 3A–C). By immunohistochemical staining, AT-1R and AT-2R expression was restricted to the innermost layer of the retina in the controls, which was increased throughout the ganglion cell layer (GCL) and inner plexiform layer of the retina following systemic hypotension (Fig. 3D, E). Increased expression of AT-1R and AT-2R was observed at the optic nerve head of systemic hypotensive rats at week 12 (Fig. 3F). These findings suggested that elevated systemic AngII levels induced by systemic hypotension led to the activation of AngII receptors and could be related to glial cell activation in the retina and optic nerve head.

### Investigation of cell death type and related pathways following systemic hypotension

**Fig. 4:**
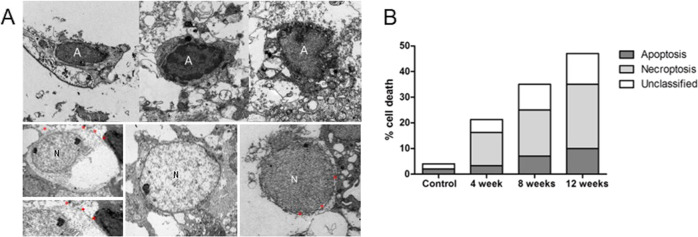
Transmission electron microscopy (TEM) of retinal ganglion cells (RGCs). **A** Cells with condensed chromatin representing apoptosis were specified as A, and cells with perinuclear space dilation and decreased electron density in the cytoplasm representing necroptosis were specified as N. **B** Discontinuous cell membrane (red asterisks), electron-lucent nucleus, and perinuclear space dilation (red asterisks) are observed in cells representing necroptosis. As the proportion of dying RGCs increased after systemic hypotension, the proportion of necroptotic cells increased. For TEM analysis, *n* = 6 for control and *n* = 6 for systemic hypotension at each time period; total *n* = 24.

The type of RGC death in the retina was investigated using TEM following systemic hypotension. Types of cell death were classified based on the RGC morphology. Apoptotic cells with condensed chromatin in the nucleus were defined as apoptosis (Fig. 4A, specified as A). Electron-dense structures were located inside the cell membrane in apoptotic cells [24]. On the other hand, the fragmented cells with discontinuous cell membrane, perinuclear space dilation, and decreased electron density in the nucleus or the cytoplasm were also observed, indicative of RGC necroptosis (Fig. 4A, specified as N) [25]. The numbers of apoptotic and necroptotic cells were quantified, and the proportion of each cell death pathway per total cells counted was assessed. For the entire duration of the experiment, there was greater proportion of necroptotic cells than that of apoptotic cells in the retinal GCL. As the proportion of dying RGCs increased following systemic hypotension, the proportion of necroptotic cells increased (Fig. 4B).

**Fig. 5:**
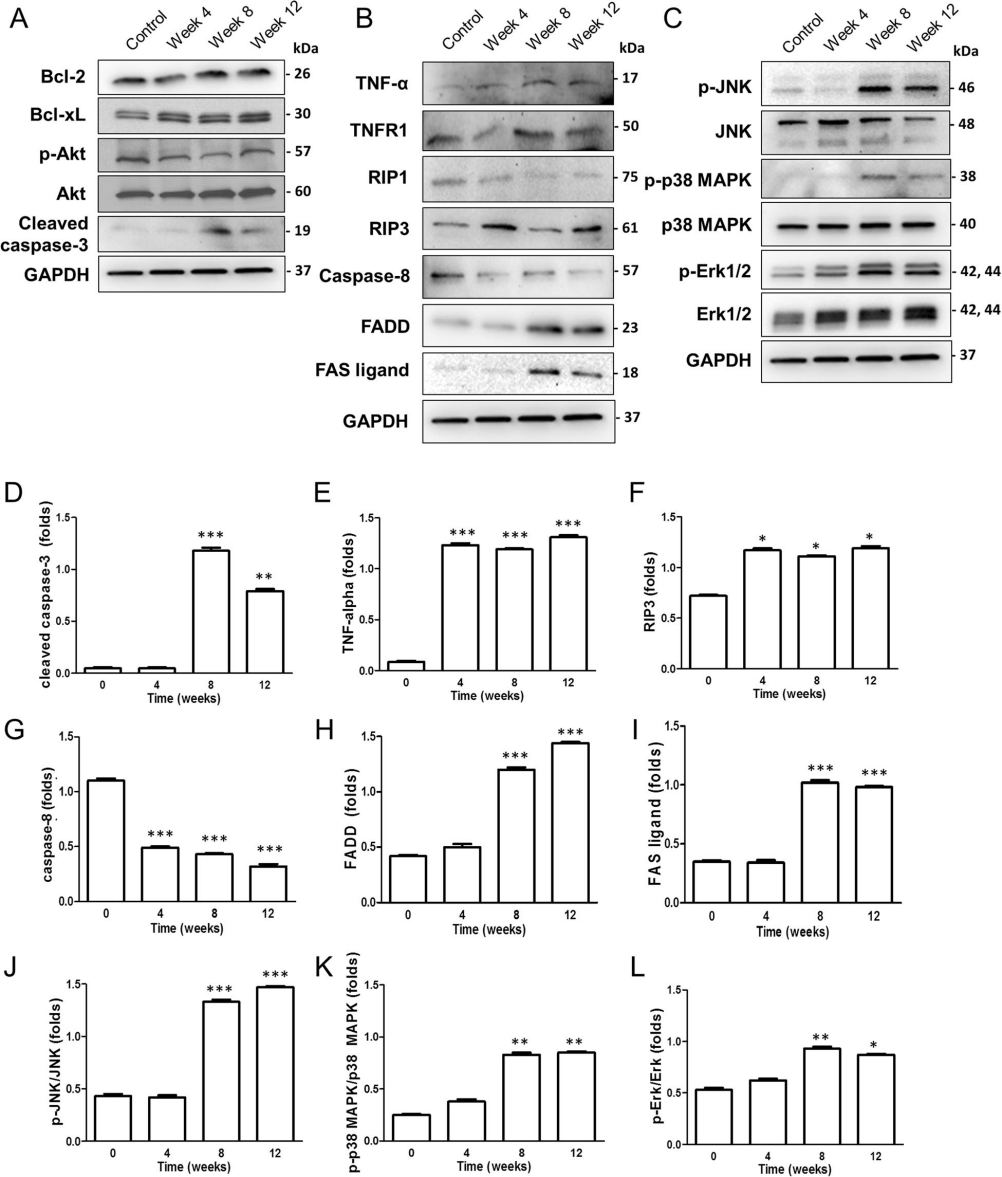
Western blot analysis of molecular pathways involved in RGC death after systemic hypotension. Western blots of (**A**) intrinsic apoptotic, (**B**) extrinsic apoptotic and necroptotis, and (**C**) protein kinase signaling pathways. Involvement of (**D**) intrinsic apoptosis, (**E**– **F**– **G**) extrinsic apoptosis and necroptosis associated with tumor necrosis factor (TNF), and (**H**, **I**) Fas were significantly altered following induction of systemic hypotension. **J**– **K**– **L** Molecules associated with kinase signaling pathway were upregulated. For western blot analysis, *n* = 6 for control and *n* = 6 for systemic hypotension at each time period; total *n* = 96. Bar represents mean ± SD. Student’s *t*-test was used for statistical evaluation. *P < 0.05, **P < 0.01, and ***P < 0.001 compared to the control.

To elucidate the molecular pathways associated with RGC death, we performed western blot analysis of the major signaling molecules involved in RGC death. Intrinsic (Fig. 5A) and extrinsic pathways of apoptosis and necroptosis (Fig. 5B), protein kinase signaling pathways (Fig. 5C) were evaluated. Among various factors, we observed elevation in cleaved caspase-3 levels suggesting involvement of apoptosis (Fig. 5D). Proteins related to extrinsic apoptosis and necroptosis pathways of TNF (Fig. 5E–G) and Fas (Fig. 5H, I) were significantly altered following systemic hypotension suggesting involvement of necroptosis. Proteins involved in kinase pathways, such as c-Jun N-terminal kinase (JNK) (Fig. 5J), p38 mitogen-activated protein kinase (p38 MAPK) (Fig. 5K), and extracellular signal-regulated kinases (Erk) (Fig. 5L), were upregulated suggesting that these pathways may be involved in glial cell activation following systemic hypotension. Other than apoptotic pathways, other proteins involved in necroptosis (especially TNF, RIP3, Fas ligand, and FADD) and JNK could be involved in RGC death following systemic hypotension.

### Comparison between ocular hypertension and systemic hypotension

**Fig. 6:**
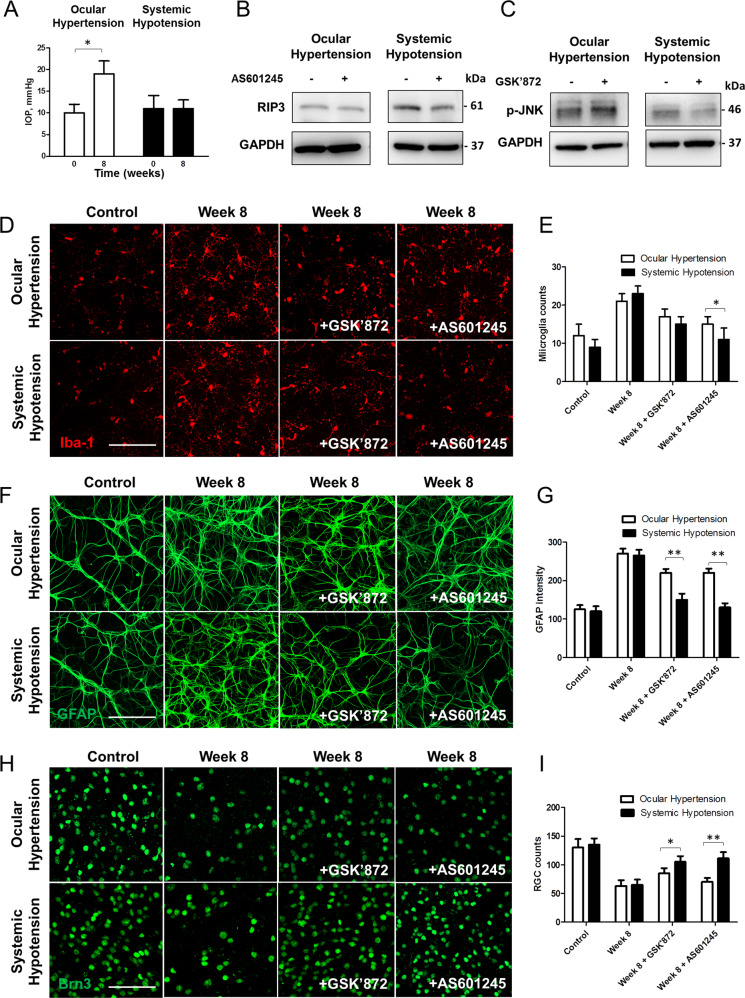
Comparisons between systemic hypotensive rats and ocular hypertensive rats. **A** Intraocular pressure (IOP) was significantly elevated after 8 weeks of experiment. **B**, **C** Western blots of RIP3 and p-JNK were performed with or without GSK’870 or AS601245 at week 8 in each model. Significant downregulation of RIP3 and p-JNK was observed only in systemic hypotensive rats when administered GSK’870 or AS601245. For western blot analysis, *n* = 6 for control and *n* = 6 for systemic hypotension at week 8; total *n* = 24. Bar represents mean ± SD. Student’s *t*-test was used for statistical evaluation. *P < 0.05 compared to the control. **D**, **F**, **H** Immunohistochemical staining and **E**, **G**, **I** western blots of Iba-1, GFAP, and Brn3a. GSK’870 or AS601245 administration made alterations of expression only in systemic hypotensive rats. Scale bars: 50 μm. For immunohistochemical staining, *n* = 6 for control and *n* = 6 for systemic hypotension at each time period; total *n* = 48. Bar represents mean ± SD. Student’s *t*-test was used for statistical evaluation. *P < 0.05 and **P < 0.01 compared to the control.

In ocular hypertensive rat, IOP was significantly elevated after 8 weeks of cauterization relative to baseline (19.2 ± 3.7 mmHg vs. 10.2 ± 2.4 mmHg; *P* < 0.001) (Fig. 6A). IOP in systemic hypotensive rat was within the normal range throughout the experimental period.

To investigate the differences in mechanisms underpinning RGC death, JNK inhibitor (GSK’872, 5 μM/kg; R&D Systems) or RIP3 inhibitor (AS601245, 20 mg/kg; Alesis Biochemicals, Lausen, Switzerland) was administered intraperitoneally to both ocular hypertensive and systemic hypotensive rats. To confirm the inhibitory effects of GSK’870 or AS601245, western blot analysis of RIP3 (Fig. 6B) or p-JNK (Fig. 6C) was performed. When compared to the retina without GSK’870 or AS601245 at week 8 in each model, significant downregulation of RIP3 and p-JNK proteins was found in the retina of systemic hypotensive rat.

Immunohistochemical staining of Iba-1 revealed similar microglial activation following ocular hypertension or systemic hypotension in the retina. However, a significant reduction in microglial activation was noted in the retina of systemic hypotensive rat compared to that in ocular hypertensive rat after intraperitoneal AS601245 injection (Fig. 6D, E). Immunohistochemical staining of GFAP revealed similar activation of astrocytes in the retinal GCL after induction of ocular hypertension or systemic hypotension. However, a significant reduction of astrocyte activation in the retina of systemic hypotensive rat was observed compared to that in ocular hypertensive rat after intraperitoneal GSK’870 and AS601245 injection (Fig. 6F, G). To examine changes of the RGCs after intraperitoneal GSK’870 or AS601245, we performed immunohistochemical staining with Brn3a in flat-mounted retina. There was similar reduction in RGC counts following ocular hypertension or systemic hypotension. However, a significant increase in RGC counts was noted after intraperitoneal GSK’870 or AS601245 in the systemic hypotensive rats but not in the ocular hypertension rats (Fig. 6H, I). These findings suggested that inhibiting necroptosis or JNK pathway attenuated glial cell activation and RGC death following systemic hypotension, but not following ocular hypertension.

## Discussion

The aim of this study was to explore the characteristics of RGC death that could occur in glaucoma cases that have unstable hemodynamic characteristics with normal range IOP. After induction of systemic hypotension in an animal model, increased AngII and its receptors were identified in the retina. Increased angiotensin levels triggered glial cell activation, which subsequently induced TNFα upregulation. We identified an increase of TNFα associated necroptotic factors such as RIP3. Levels of inactive caspase 8, which is an inherent necroptosis inhibitor [26], were decreased in the retina of hypotensive animal model. Using electron microscopy, the proportion of necroptotic cell death increased gradually as the duration of hypotension prolonged. When this hemodynamic unstable retinas were compared with retinas with elevated IOP, inhibition of necroptotic pathway or JNK pathway attenuated glial cell activation and RGC death in systemic hypotensive rat, but not in ocular hypertensive rat. Collectively, these findings suggest that systemic hypotension has an involvement of distinct cell death mechanisms associated with AngII and glial cell activation.

Glaucoma with normal range IOP is characterized by a high frequency of systemic vascular dysregulation [27–29]. In a population-based study in Korea, where most glaucoma patients have normal IOP, it was reported that fluctuations in systolic BP were associated with the development of open-angle glaucoma [30]. Fluctuations in systolic BP could suggest hemodynamic instability of the patients. Therefore, we tried to make an animal model with characteristics of glaucoma with unstable BP, but with normal range IOP. This systemic hypotensive rats were designed to induce RAAS imbalance and hemodynamic instability. Systemic hypotension was induced by taking oral diuretics in order to circumvent unwanted effects on AngII receptors. From this systemic hypotensive rats, in short, BP rats, we confirmed gradual decrease in RGC number similar to that observed in other animal models of glaucoma.

AngII increase due to hemodynamic instability and subsequent RAAS functioning have been studied in a wide variety. RAAS involvement in neuroinflammation cascades and glial cell activation has been reported in neurodegenerative diseases [31]. In the retina, angiotensin receptors were also identified, and RAAS associated neuroinflammation could occur [15]. In this study, AngII levels increased following systemic hypotension, and the resultant glial cell activation indicated by upregulation of GFAP and Iba-1 suggested the occurrence of retinal neuroinflammation. Glial cell activation and upregulation of TNFα, which is the mediator of neuroinflammation [32, 33], may induce necroptosis cascade [34]. Recently, Chen et al. reported that TNFα from microglia-mediated necroptosis after ischemic brain injury [35]. The neuroprotective effects of necrostatin via necroptosis inhibition through TNFα related pathway have also been reported in subarachnoid hemorrhage [36]. Our results showed an increase in TNFα/RIP3 and decrease in inactive caspase 8, which resulted in an increase of necroptotic RGC counts following systemic hypotension. Although glaucoma is traditionally considered as a disease that represents RGC death by apoptosis, necroptosis cascades initiated by TNFα from glial activation could also play a role in RGC death in hemodynamically unstable glaucoma patients. A previous study reported the involvement of AngII in the activation of retinal microglia after exposure to hypoxia [37]. This study suggested that ocular diseases such as retinopathy of prematurity or diabetic retinopathy which has ischemic changes in the retina may have AngII as the underlying mechanism for glial cell activation. We demonstrated that systemic hypotension resulting in blood flow instability to the retina or optic nerve head, other than ischemia in the retina, may have contributed to RAAS and glial cell activation. This has important clinical relevance, since disease progression in glaucoma patients with systemic hemodynamic instability or systemic hypotension may not be stopped by ocular hypotensive treatments. Novel treatment strategies targeting glial activation and resultant neuroinflammation or necroptosis may be required for this subpopulation of glaucoma patients. Likely, glaucoma patients with systemic hypertension could present as BP instability and RAAS activation. Continuously and excessively increased angiotensin II level may be one of several pathophysiologic mechanisms that make systemic hypertension and cause sequential target organ damage. It is reported that retinal microglia expresses angiotensin II receptor, and the activated state of microglia was affected by the angiotensin level. It is possible that increased angiotensin II level in both hypertension and hypotension could explain the similar results of microglia activation.

Microglia and astrocytes constitute glia in the central nervous system, including the retina. Activated astroglial cells are termed damage-associated astroglia, since these cells play an important role in inflammatory responses and associated cell death [38]. Since damage-associated astroglial cells play an important role in inflammatory response and resulting cell death in the central nervous system, it is especially termed neuroinflammation. There were abundant previous studies showing that neuroinflammation is important in the RGC loss in glaucoma. However, this study is the first to evaluate the relationship between systemic hypotension and neuroinflammation. Additionally, we compared neuroinflammation and its role in RGC loss between elevated IOP and systemic hypotension, which are the two main mechanisms of glaucoma found in glaucoma patients. For glaucoma with elevated IOP, previous reports have suggested that oxidative stress could induce neuroinflammation and subsequent apoptotic RGC death [39]. The associated cell death pathways were accompanied by NF-κB activation [40]. These findings are distinct to our study, which targeted glaucoma with hemodynamic instability, in that our results indicated that necroptotic RGC death and the JNK pathway play major roles. Understanding the different mechanism for RGC loss in glaucoma by the underlying cause and clinical phenotype may be important to manage glaucoma patients and personalized treatments. The first step in glaucoma development may be elevated IOP. However, now we understand that cause of glaucoma is complex and there are also additive risk factors for disease progression in patients undergoing ocular hypotensive treatment or in glaucoma patients with normal range IOP.

In conclusion, this study demonstrated that systemic hypotension could trigger AngII associated glial cell activation. Neuroinflammation by activated glia induced TNFα level elevation, which resulted in RGC necroptosis in the retina. When compared to ocular hypertensive rat, the role of AngII and glial cell activation to RGC loss were important in the retina of systemic hypotensive rat. Therefore, neuroinflammation triggered by systemic hypotension and necroptotic RGC death pathway may have additional effects aside from traditional apoptotic pathway caused by elevated IOP. These results highlight the need to identify the causes of RGC loss and underlying mechanisms to optimize management of glaucoma patients and personalize treatment strategies to prevent glaucoma-associated blindness.

## Supplementary information

Raw data of Western blots

Reproducibility checklist

## Data Availability

All data generated or analyzed during this study are included in the main text and the supplementary information files.
