# Supplementary material for: Angiotensin II related glial cell activation and necroptosis of retinal ganglion cells after systemic hypotension in glaucoma
Source: Cell Death Dis. 2022 Apr 9;13(4):323. doi: 10.1038/s41419-022-04762-4 (PMC8993868; doi:10.1038/s41419-022-04762-4)

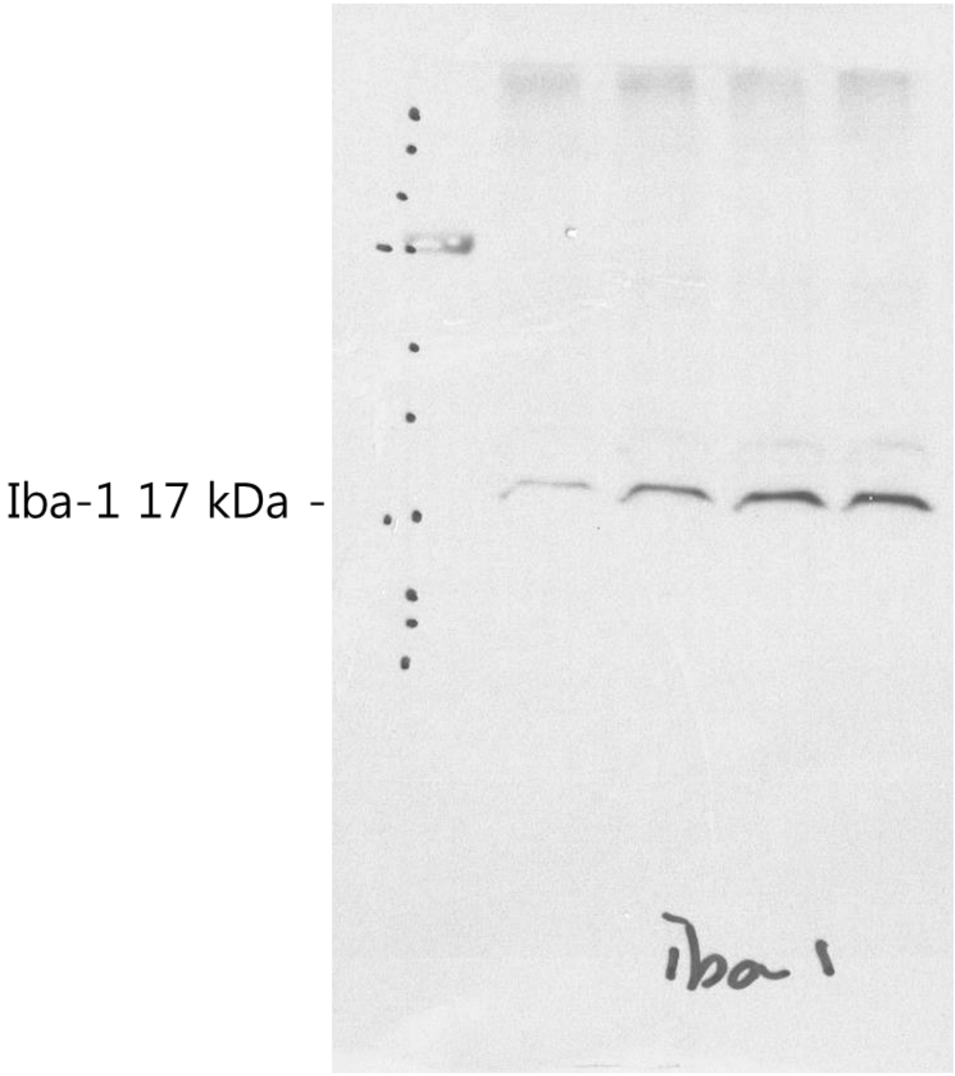
Iba-1 17 kDa -
Iba 1

AT1R 41 kDa -

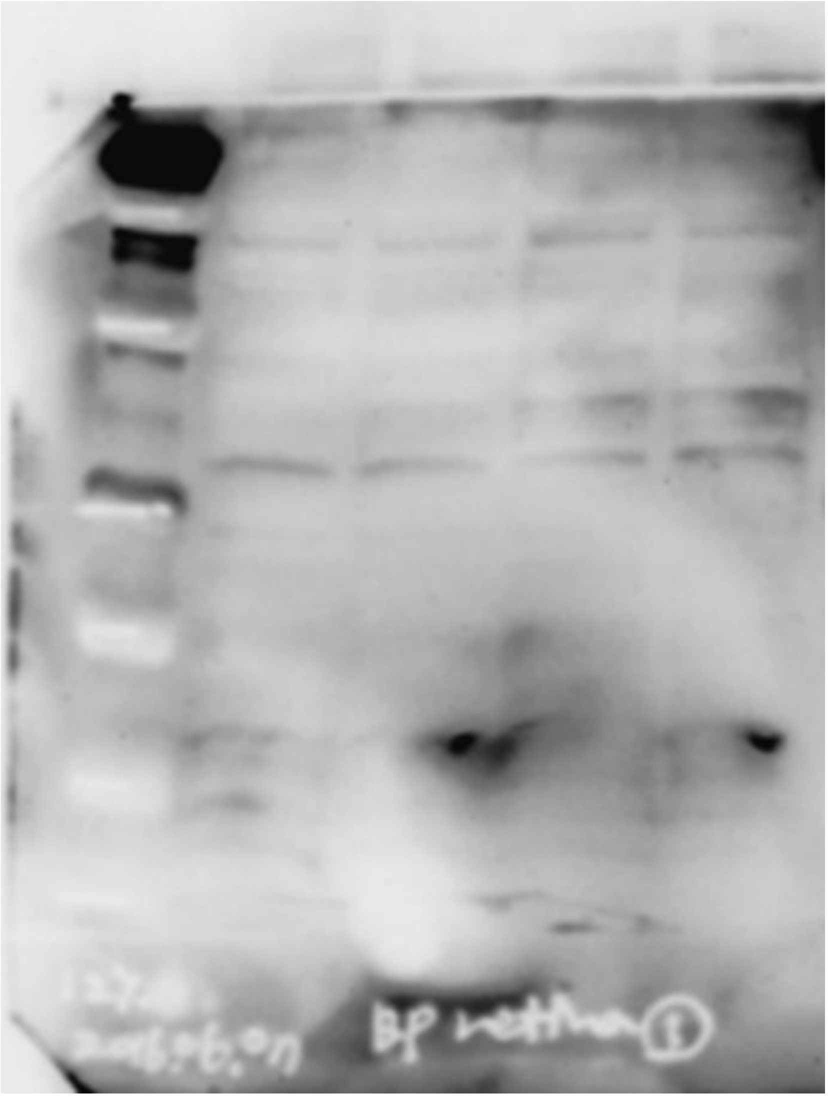

AT2R 41 kDa -

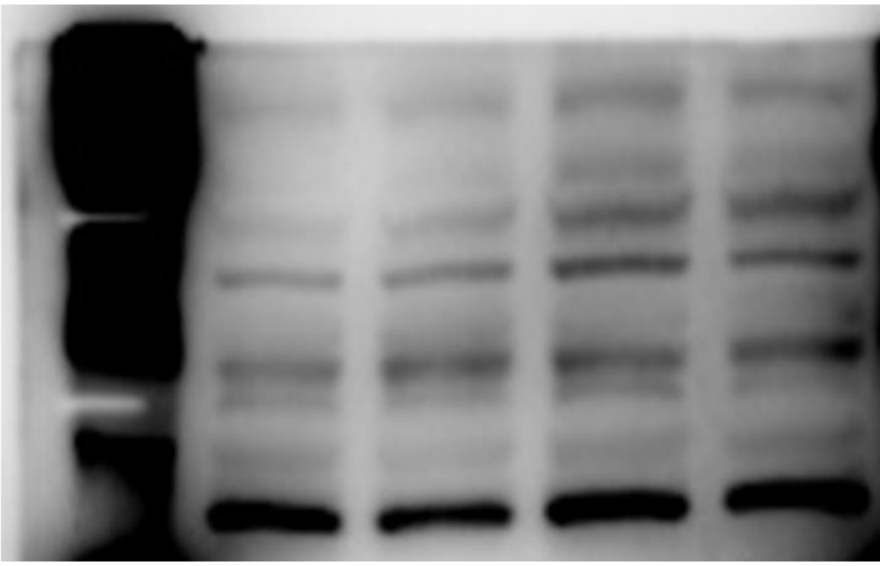

Bcl-2 26 kDa -

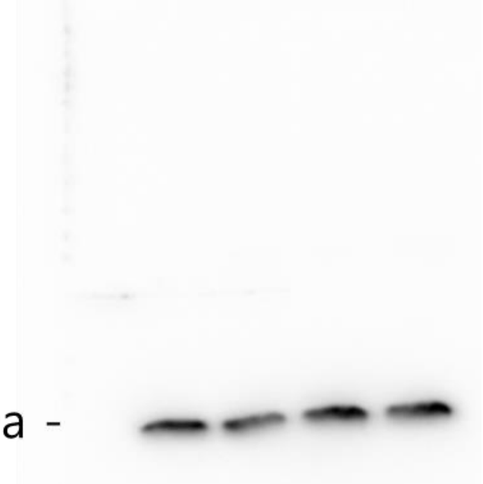

Bcl-xL 30 kDa -

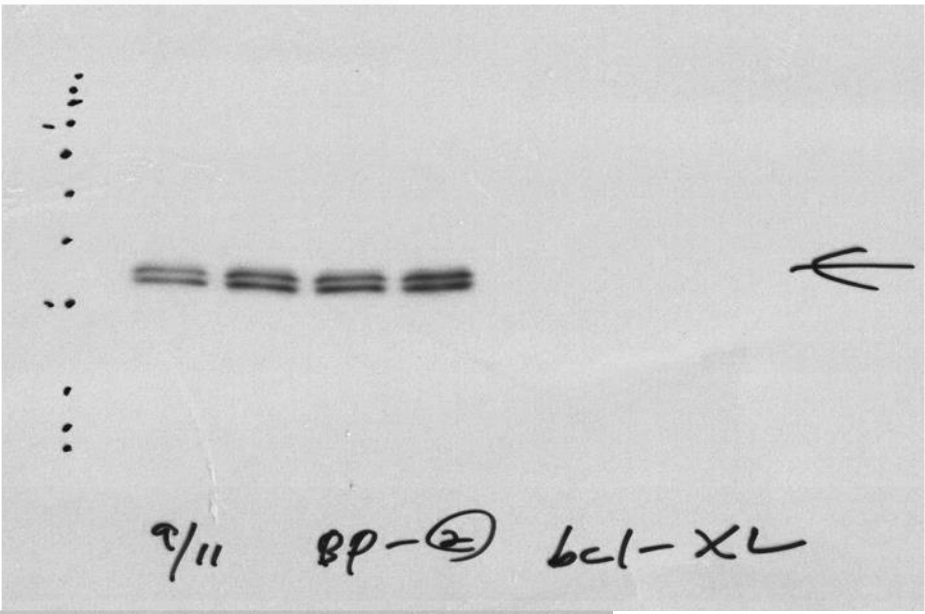

pAkt 57 kDa -

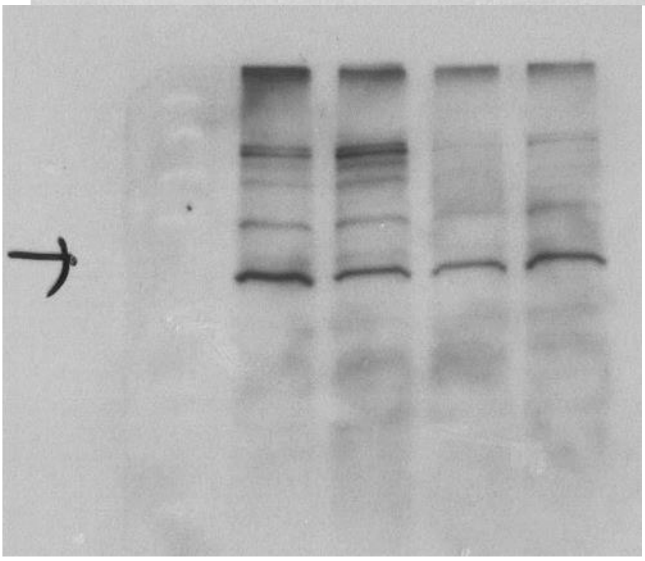

Akt 60 kDa -

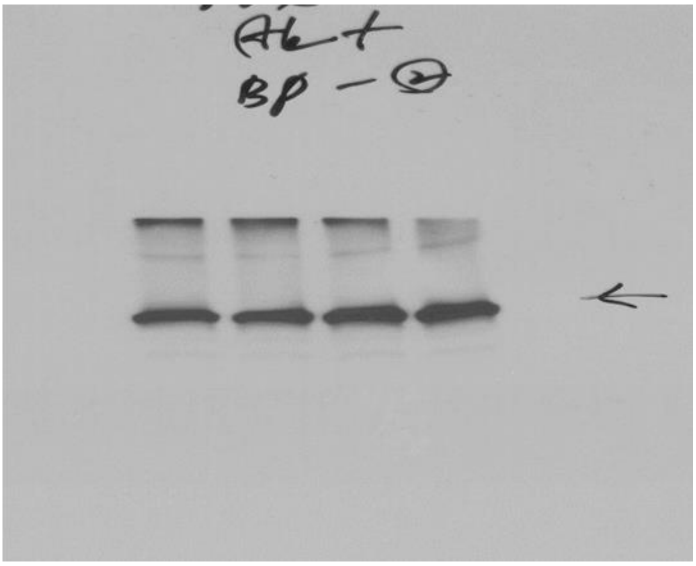

Cleaved
casapase-3
17, 19kDa -

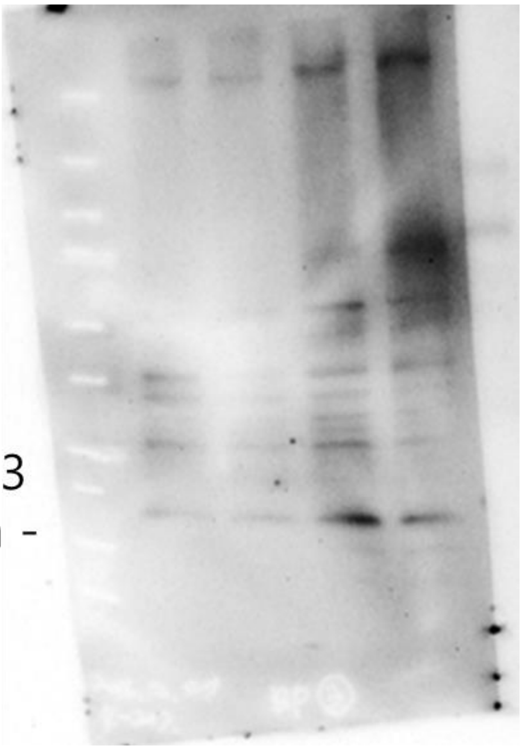

TNFα 17 kDa -

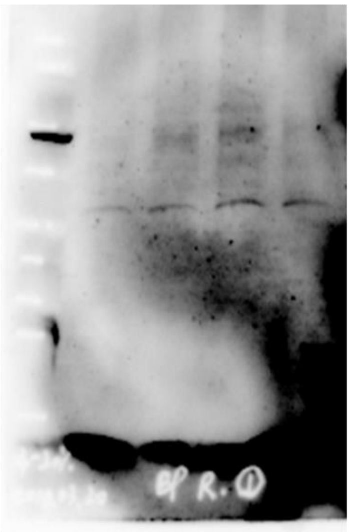

TNF R1 50 kDa -

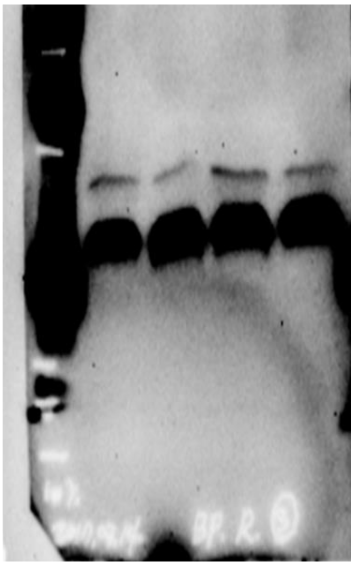

RIP1 75 kDa -

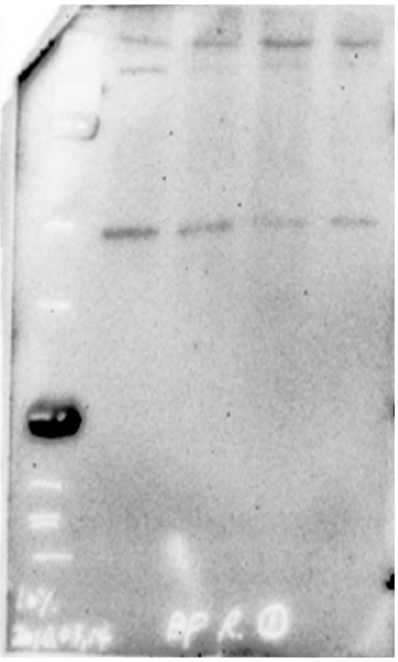

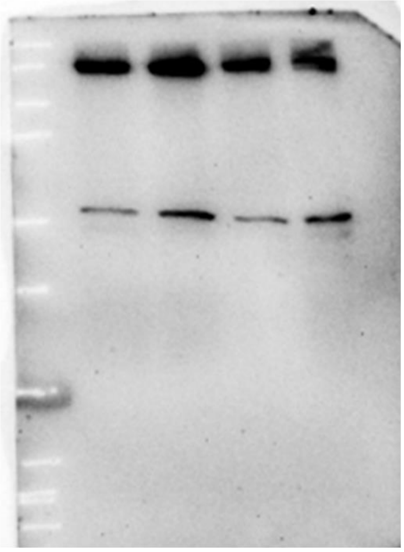

- RIP3 61 kDa

Caspase-8 57 kDa -

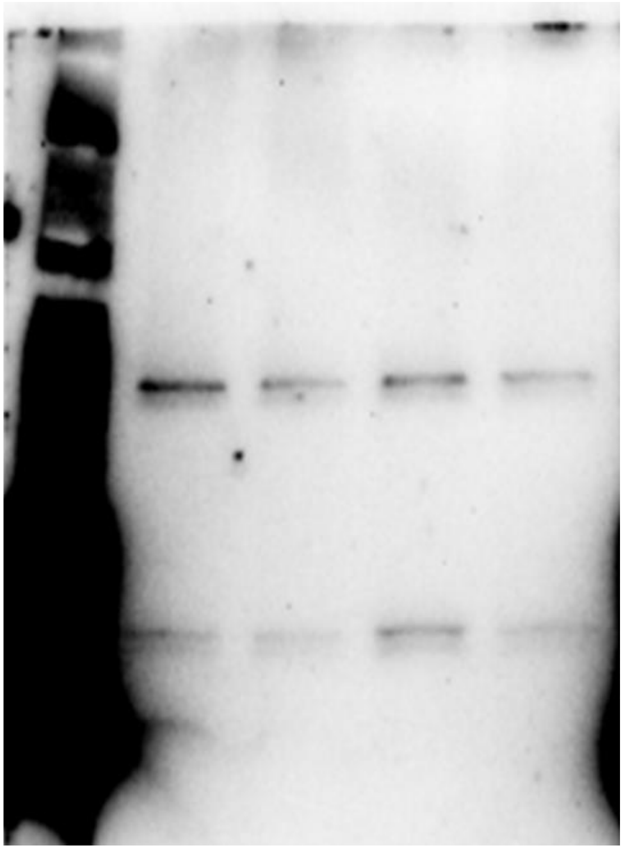

FADD 23 kDa -

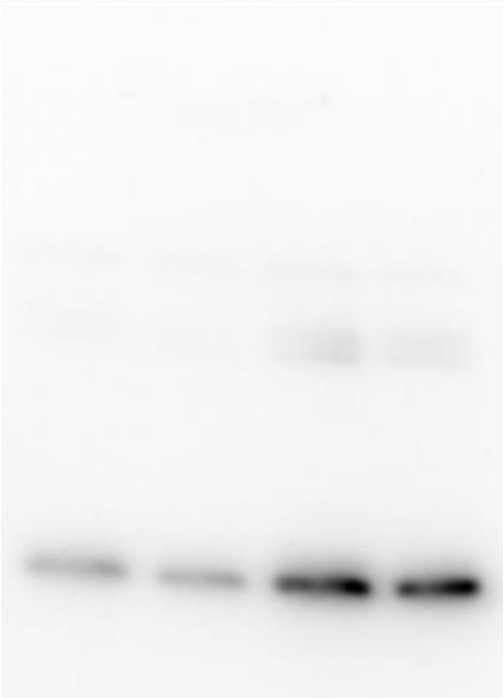

Fas ligand 18 kDa -

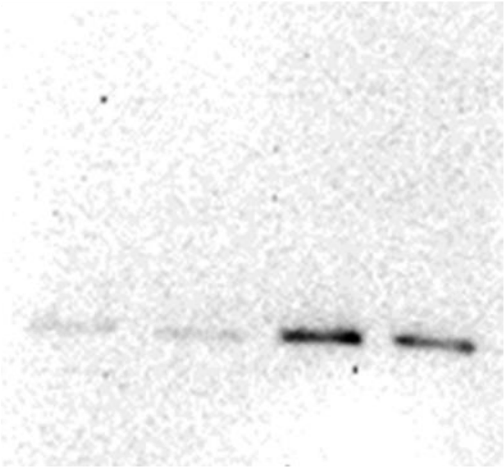

P-JNK 46 kDa -

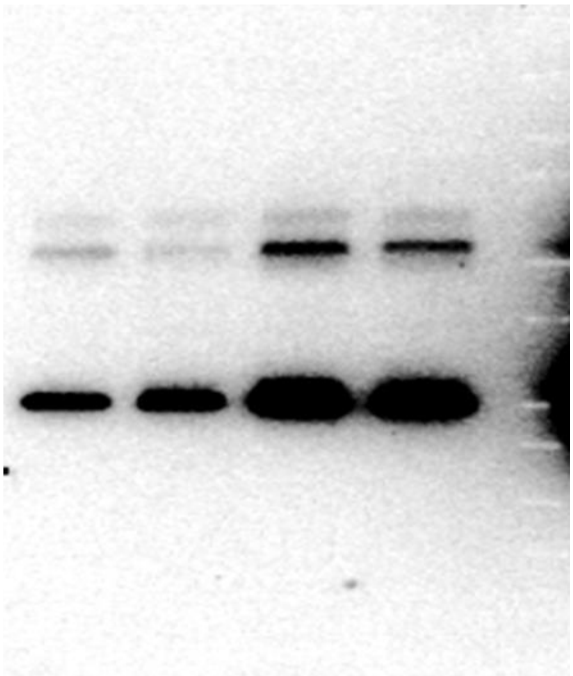

JNK 48 kDa -

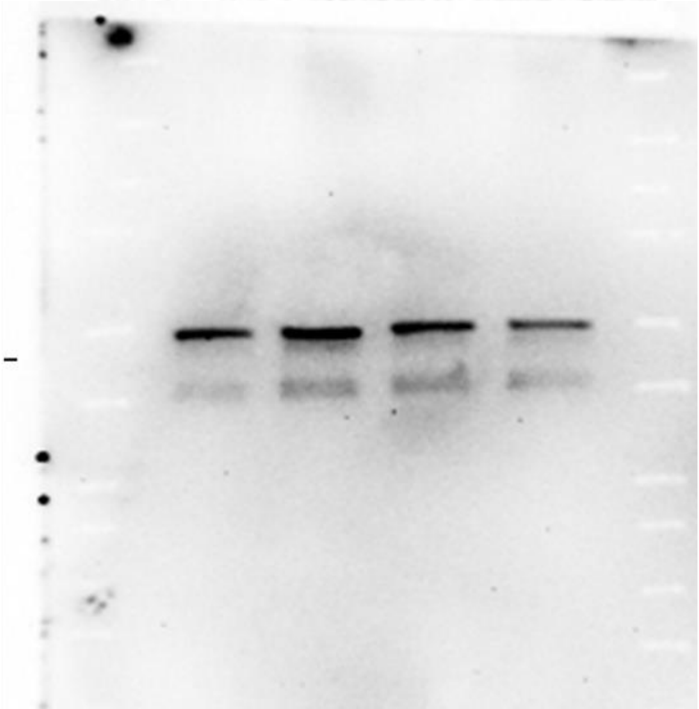

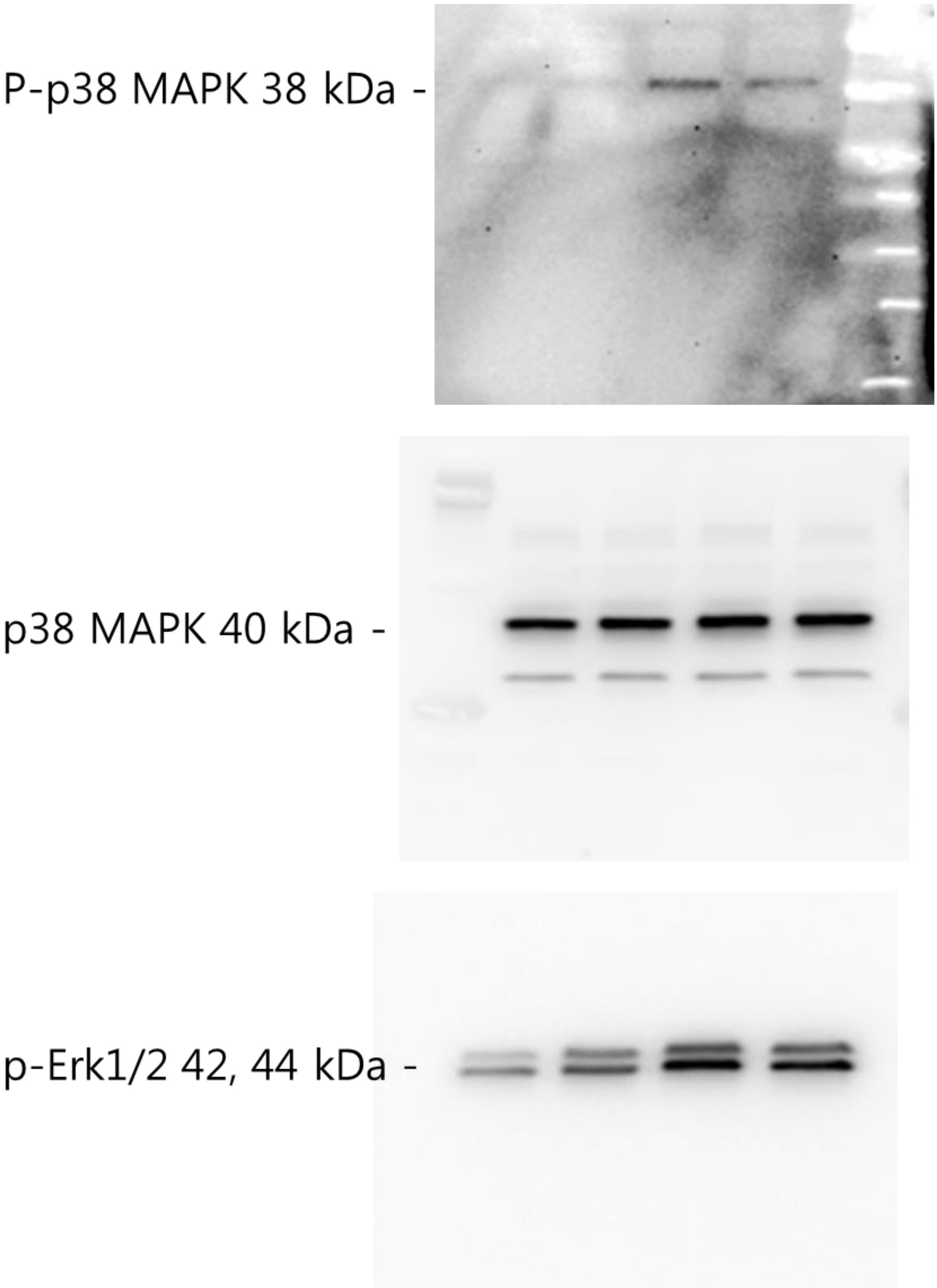
P-p38 MAPK 38 kDa -
p38 MAPK 40 kDa -
p-Erk1/2 42, 44 kDa -

Erk1/2 42, 44 kDa -

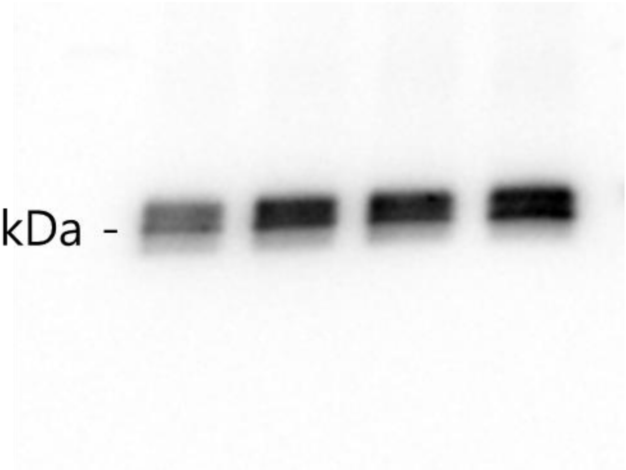

Supplement: Supplementary file 1 — Raw data of Western blots [file 41419_2022_4762_MOESM1_ESM.pdf]
